# Hepatic Metastases of Granulosa Cell Tumour of the Ovary

**DOI:** 10.1155/1996/13452

**Published:** 1996

**Authors:** José I. Rodríguez García, Juan. J. González González, Luis J. García Flórez, Paloma Floriano Rodríguez, Enrique Martínez Rodríguez

**Affiliations:** Department of Surgery B and Pathology Hospital Central Universidad de Oviedo Spain

## Abstract

A case of metastatic granulosa cell tumour of the ovary is reported. Investigations revealed a secondary tumour in segment VI and VII of the liver. Right hepatic resection was performed. Microscopic findings revealed a tumour with histological features identical to that removed eleven years before.


*HPB Surgery*, 1996, Vol.10, pp. 55–57
Reprints available directly from the publisher
Photocopying permitted by license only

© 1996 OPA (Overseas Publishers Association)
Amsterdam B.V. Published in The Netherlands
by Harwood Academic Publishers GmbH
Printed in Malaysia

### CASE REPORT
### Hepatic Metastases of Granulosa Cell Tumour of the Ovary

JOSÉ I. RODRÍGUEZ GARCÍA, JUAN. J. GONZÁLEZ GONZÁLEZ, LUIS J. GARCÍA FLÓREZ, PALOMA FLORIANO RODRÍGUEZ and ENRIQUE MARTÍNEZ RODRÍGUEZ

Department of Surgery B and Pathology, Hospital Central. Universidad de Oviedo, Spain

*(Received 3 October 1995)*

A case of metastatic granulosa cell tumour of the ovary is reported. Investigations revealed a secondary tumour in segment VI and VII of the liver. Right hepatic resection was performed. Microscopic findings revealed a tumour with histological features identical to that removed eleven years before.

KEY WORDS: Liver metastasis hepatic metastasis ovarian neoplasms

### INTRODUCTION

The natural history of many malignant tumours, and therefore their metastatic pattern, has yet to be defined. The liver is frequently the seat of metastases, indeed, they appear either synchronously or metachronously in about 40% of cases[1]. At present, the surgical treatment of hepatic metastases is limited almost exclusively to those of colorectal origin; it is more difficult to establish thereapeutic guidelines for less common tumors unless these occur in an advanced stage of the illness, as the benefit likely to result from surgery, or from associated or alternative procedures, is unknown. In such situations, apart from increased survival, alleviation of symptoms takes on particular importance. In the present case we report our experience with a patient having a single metachronous metastasis of a granulosa cell tumour of the ovary.

### CASE REPORT

A 62 year old woman was referred to our Department after physical examination demonstrated hepatomegaly, and an ultrasound study revealed the presence of a hepatic mass. Noteworthy in her clinical history was a total hysterctomy with bilateral adnexectomy, due to a granulosa cell tumour of the ovary, eleven years previously. She was subsequently given radiotherapy treatment (Co 60, 22 Gy) and chemotherapy (Melfalan). Chemotherapy had to be suspended because of thrombocytopenia. Six years after the operation, ultrasound revealed a 5 × 5 cm. mass in the right hepatic lobe. A dynamic CT scan and hepatic arteriography were performed, revealing an 11.5 × 10.5 cm. hypervascular mass affecting segments VI and VII of liver (Figure 1). These findings were verified at surgery, with no evidence of abdominal dissemination, and a right hepatectomy was carried out. Transitory ascites and jaundice were present in the postoperative period. In the pathological study (Figure 2) a tumour with histological features identical to that removed in 1980 was found. The patient died 9 months later of cerebrovascular accident without tumour recurrence at autopsy.

### DISCUSSION

Ovarian tumours are responsible for 5–6% of cancer deaths among women. Granulosa cell tumour represents about 2% of ovarian tumours and is accompanied by hepatic metastases in 4% of cases[2-4], usually

*Correspondence to*: Dr. Juan J. GonzálezValentín Masip 5-6-[2]E 33013 Oviedo, Spain

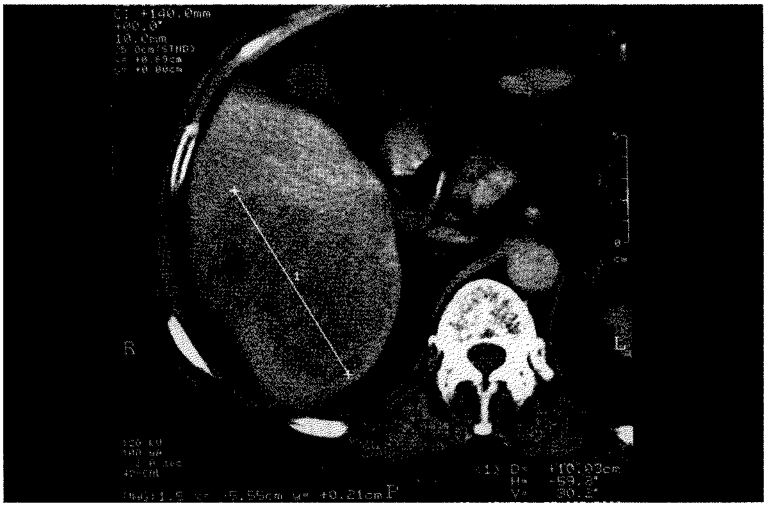

**Figure 1** CT scan. Hepatic mass in segments VI and VII.

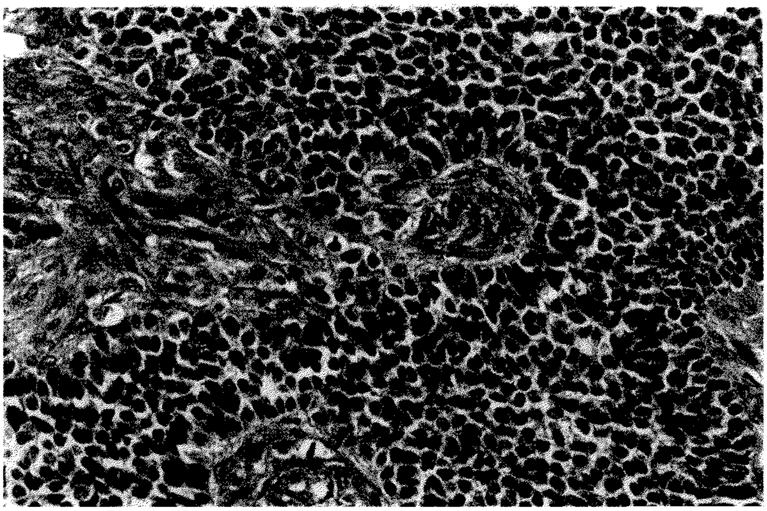

**Figure 2** Hepatic metastases of granulosa cell tumour of the ovary. Call-Exner's bodies Typical coffée-grain. H-E. 200X.

being multifocal and occupying a wide area of the hepatic parenchyma[5]. Clinically, these metastases may appear as hepatomegaly, abdominal mass, compression of neighbouring organs, or, characteristically, in intraabdominal haemorrhage, with high morbidity and mortality[5].

Ultrasonography, CT scan and/or angiography may be used in topographic diagnosis. The histological nature can be ascertained by fine needle aspiration biopsy, although differential diagnosis is sometimes difficult with metastases of adenocarcinoma, cystadenocarcinoma, carcinoid, hepatocellular carcinoma, Brenner's tumour or Sertoli's cell tumour[1].

The difficulty of obtaining an accurate preoperative diagnosis, the incomplete and transitory response of these tumours to radiotherapy and to various chemotherapeutic agents, and the possibility of complications resulting from intratumoral bleedinng lead us to believe that surgical treatment is justified when the extent and location of the tumour permit, a circumstance which has not so far been reported.

### REFERENCES

1. Edmondson H.A., Peters R.L.(1982) Neoplasms of the liver. In: Diseases of the liver, 5th edn, edited by Shiff ER, pp. 1101–1157. Philadelphia: Lippincot.

2. Dvoretsky P.M., Richards K.A., Angel C., Rabinowitz L., Stoler M.H., Beecham J.B., Bonfiglio T.A. (1988) Distribution of disease at autopsy in 100 women with ovarian cancer. *Human Pathology* **19**: 57–63.
3. Fox H., Agrawal K., Langley F.A. (1975) A clinicopathologic study of 92 cases of granulosa cell tumor of the ovary with special reference to the factors influencing prognosis. *Cancer* **35**: 231–142.
4. Rose P.G., Piver M.S., Tsukada Y., Lau T. (1989) Metastatic patterns in histologic variants of ovarian cancer. An autopsy study. *Cancer* **64**: 1508–1513.
5. Margolin K.A., Pak H.Y., Esensten M.L., Doroshow J.H. (1985) Hepatic metastasis in granulosa cell tumor of the ovary. *Cancer* **56**: 691–695.
6. Benda J.A., Zaleski S. (1988) Fine needle aspiration cytologic features of hepatic metastasis of granulosa cell tumor of the ovary. Differencial diagnosis. *Acta Cytologica* **32**: 527–532.

### *Commentary to paper "Hepatic metastasis of granulosa celltumour of the ovary"*

The value of hepatic resection in the treatment of liver metastasis is well established in, for example colorectal metastasis. There is also a general agreement on prognostic factors in this group identifying extent of liver evolvement, extrahepatic intraabdominal growth and resection margins which have special importance for long term survival. There is however no consensus regarding treatment of hepatic metastasis for other organs and especially not from tumours outside the non portal area. Liver metastases from ovarian cancers are rare and especially granulosa cell tumours. This group of patients is therefore rarely considered for liver resection. Especially since most appear multiple and extremely rarely are single. Overall it is noted that the liver is involved in more than 50% of the patients with ovarian cancer and it is likely that the majority of these metastases are local extensions from peritoneal surfaces and not really the result of vascular dissemination to the liver. The route of dissemination in this case is hard to elucidate, but since the disease in this case did not seem to follow the usual trend an aggressive surgical approach might be worthwhile. This was proven in this situation and we should be reminded of this possibility in patients with liver metastasis from ovarian tumours.

Dr. B W Jeppsson
Department of Surgery
Lund University
LUND
S-221 85
SWEDEN

